# A rare case of polymicrobial brain abscess involving *Actinomyces*

**DOI:** 10.1016/j.radcr.2021.02.042

**Published:** 2021-03-04

**Authors:** Abdelrhman Abo-Zed, Mohamed Yassin, Tung Phan

**Affiliations:** a Department of Medicine, University of Pittsburgh Medical Center, Pittsburgh, PA, USA; b Division of Clinical Microbiology, University of Pittsburgh and University of Pittsburgh Medical Center, 3477 Euler Way, Pittsburgh, PA 15213, USA

**Keywords:** Brain, Abscess, Polymicrobial, Actinomycosis, VITEK 2

## Abstract

Brain abscess is a focal intracranial infection that may present as a life-threatening emergency. Brain abscess can present with a wide range of clinical syndromes, and it results in high morbidity and mortality worldwide. Here we describe a rare case of the polymicrobial right parietal brain abscess, especially associated with *Actinomyces* in a 59-year-old male who presented with acute onset of left-sided weakness and altered mental status. The patient underwent surgical treatment (burrhole aspiration) and antibiotics with good improvement. Prompt diagnosis and treatment are critical for brain abscess and ultimately lead to better patient outcomes.

## Introduction

Brain abscess is a serious life-threatening condition. This disease may lead to permanent sequelae although there have many advances in diagnosis and management [1]. Brain abscess can present with a wide range of clinical syndromes depending on the size, location, and characteristics of the abscess [2]. Incidence is estimated at 0.4 to 1.3 per 100,000 people per year and increased in immunosuppressed patients [3]. Brain abscess typically results from a predisposing factor as neurosurgical procedures or trauma. Additionally, contiguous spread from areas surrounding the brain (such as mastoiditis, sinusitis, and dental infection) and hematogenous dissemination are responsible for a significant percentage of brain abscesses [4,5]. HIV infection and other causes of immunosuppression are often associated with brain abscess caused by opportunistic infections [6]. In immunocompetent patients, bacteria are responsible for at least 95% of brain abscesses [7]. Here we report a case of polymicrobial right parietal brain abscess, especially associated with *Actinomyces* in an immunocompetent individual.

## Case presentation

**Fig. 1:**
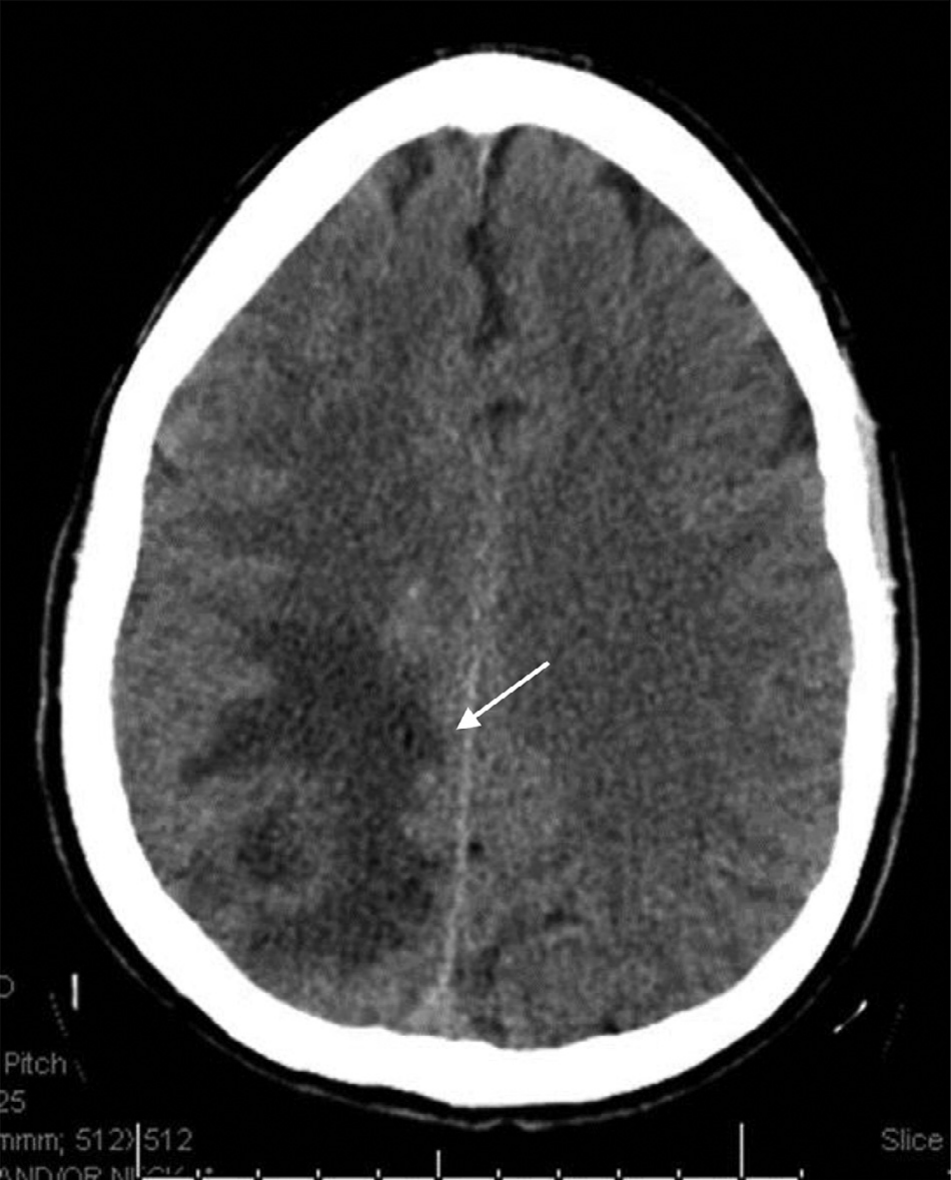
The CTA of the brain. The cut provided is a post contrast cut showing a large peripherally enhancing mass centered within the right parietal lobe measuring approximately 2.5 × 2.7 cm with surrounding vasogenic edema and localized mass-effect with a 0.6 cm right to left midline shift.

**Fig. 2:**
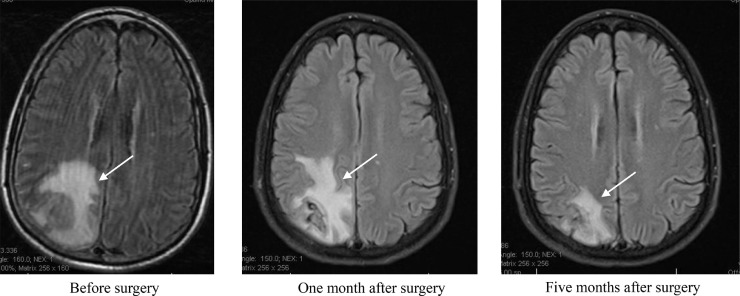
The MRI of the brain. The first MRI (before surgery) with noncontrast confirmed the right parietal rim-enhancing lesion. The second and third MRI follow-up (with and without contrast) showed significant improvement in size and surrounding vasogenic edema. All the provided images are axial T2 Flair.

A 59-year-old male with a medical history of diverticulosis, chronic back pain, presented to the emergency room with acute onset of left sided weakness and altered mental status. His wife noted that he was getting gradually confused over the past few days prior to admission. On admission, the patient was afebrile and vitally stable. On examination, he had left-sided hemiparesis, confusion and anisocoria. Stat computed tomography head and angiogram (CT and CTA) revealed a large peripherally enhancing mass within the right parietal lobe measuring 2.5 × 2.7 cm with vasogenic edema and localized mass-effect with a 0.6 cm right to left midline shift (Fig. 1). The CT scan also showed advanced periodontal disease. After immediate return from the CT scanner, the patient developed a focal seizure with secondary generalization that was controlled with multiple doses of lorazepam intravenously and maintenance antiepileptics. The patient was intubated for airway protection. Laboratory evaluation was also significant for a leukocytosis of 15.5 with 89.1% neutrophils and 5.6% lymphocytes, but otherwise unremarkable. Magnetic resonance imaging of the brain confirmed the right parietal rim-enhancing lesion with central marked diffusion restriction (Fig. 2). Stereotactic aspiration of the abscess was performed via a right parietal burr hole craniotomy, and approximately 5 mL of purulent fluid was aspirated.

The brain abscess aspirate collected from this patient during the surgery was submitted to the clinical microbiology laboratory for fungal and bacterial cultures. No fungal growth was noted on the Sabouraud dextrose agar. The bacterial culture grew multiple microorganisms including *Actinomyces, Fusobacterium, Peptostreptococcus*, and *Diphtheroid*. The recommendations for management included 6 weeks of intravenous meropenem and vancomycin. The patient's symptoms markedly resolved, and he had 2 MRI follow-up with resolution of abscess (Fig. 2). He was continued on prophylaxis antiepileptics and 6 months of oral doxycycline for suppression.

## Discussion

**Fig. 3:**
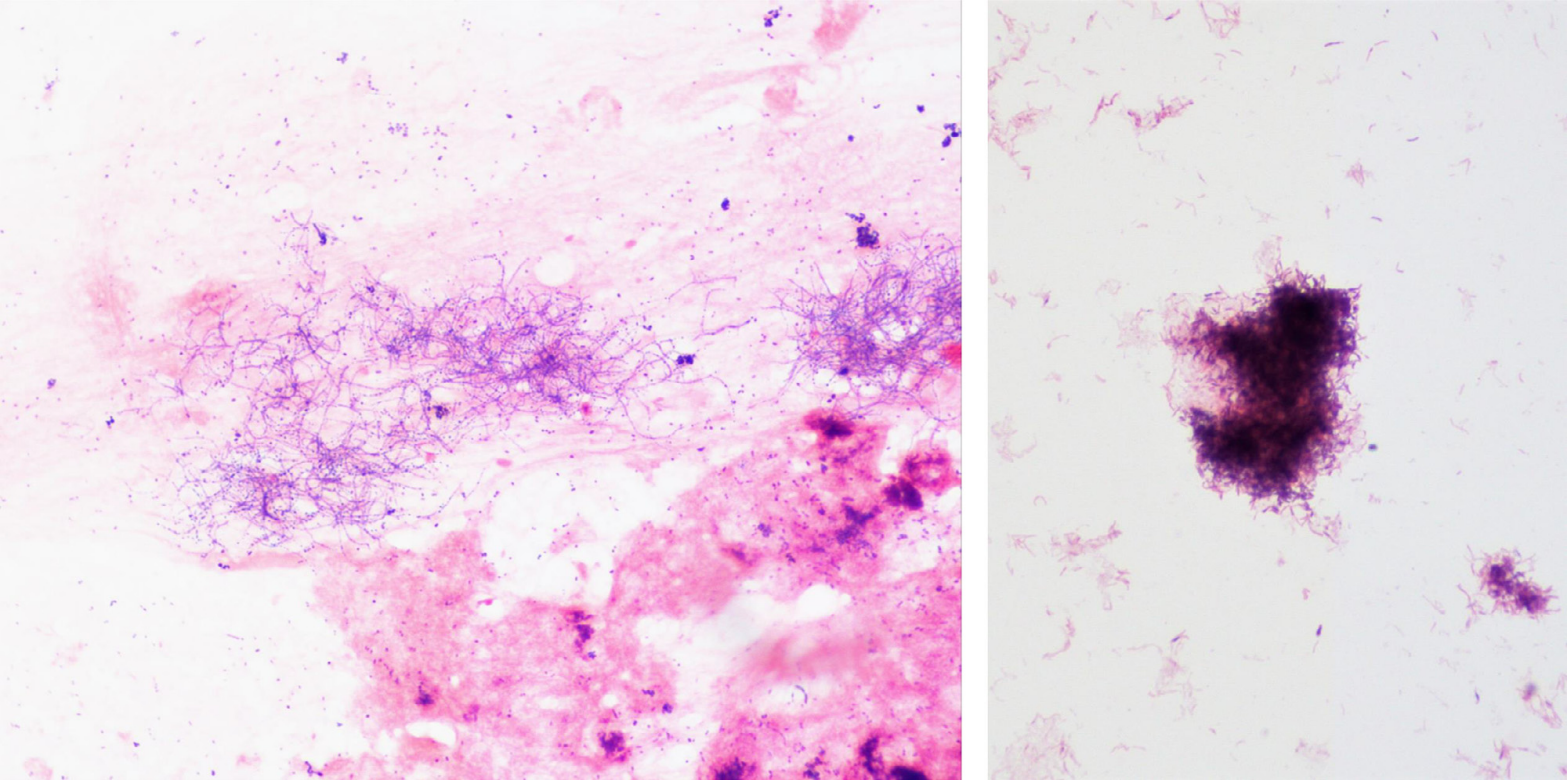
Microscopic examination of a gram-stained smear revealed that the microorganism grew in clusters of tangled filaments and had the typical morphology of Actinomyces as branching, filamentous, and beaded gram-positive bacilli at 1000× magnification.

Brain abscess is a serious infection that is suspected clinically with high-risk predisposing conditions and radiographic imaging. It can be associated with death or devastating permanent neurologic deficits [8,9]. In our case, the patient is immunocompetent and did not have any predisposing conditions. The multiple microorganisms involved are highly suggestive of oral infection, Although the patient had a pilonidal cystectomy 2 weeks prior to admission, there were no cultures obtained and no previous association in literature. Actinomycosis is an infection by a species within the genus *Actinomyces*, generally seen in dental and other oropharyngeal abscess formations. It is well-established that actinomycosis is an endogenous infection. However, central nervous system involvement is very rare [10,11]. In this case, microscopic examination of a gram-stained smear revealed that the microorganism grew in clusters of tangled filaments and had the typical morphology of *Actinomyces* as branching, filamentous, and beaded gram-positive bacilli (Fig. 3). *Actinomyces* is known as a commensal of mucosal membranes of the oropharyngeal cavity. Its infection begins with a breach of the mucosa and is associated with poor oral hygiene, preexisting dental disease, or dental procedure [12,13]. In addition, the infection is often polymicrobial and can be associated with other oral bacteria such as *Fusobacterium, Peptostreptococci* [12,14] as seen in our case. The CT scan revealed multifocal apical rarefying osteitis in the background of advanced periodontal disease. It is likely that the advanced periodontal disease is associated with the polymicrobial brain abscess since the patient did not have any other potential predisposing conditions.

Here we present a rare case of right parietal brain abscess associated with *Actinomyces* in an immunocompetent patient. Our study emphasized the importance of prompt radiological identification and immediate proper treatment of brain abscess to avoid complications.

## Ethical approval/Patient consent

Approval from the ethical committee was not required due to the nature of this case report. Abiding by the Declaration of Helsinki, patient anonymity was guaranteed.
